# Comparison of outcomes of trans-subclavian versus trans-apical approaches in transcatheter aortic valve implantation

**DOI:** 10.1186/s13019-022-01929-0

**Published:** 2022-08-04

**Authors:** Olaf Tomala, Vipin Zamvar, Rong Bing, Renzo Pessotto, Nick Cruden

**Affiliations:** 1 Department of Cardiothoracic Surgery, Royal Infirmary of Edinburgh, Edinburgh, EH16 4SA UK; 2 Department of Cardiology, Royal Infirmary of Edinburgh, Edinburgh, UK

**Keywords:** Transcatheter aortic valve implantation, Vascular access, Aortic stenosis, TAVR

## Abstract

**Background:**

Many patients are unsuitable for conventional femoral transcatheter aortic valve implantation (TAVI) but there is limited evidence as to which alternative approach has the best outcomes. We compared clinical outcomes in patients undergoing trans-subclavian (TS) or trans-apical (TA) TAVI.

**Methods:**

This was a national retrospective observational study of patients undergoing surgical TAVI in Scotland between January 2013 and March 2020. The pre-operative patient characteristics, intraoperative details and post-operative outcomes were compared between TS and TA cohorts using data from the National Institute of Cardiovascular Outcomes Research (NICOR) registry.

**Results:**

Among 1055 patients who underwent TAVI, TS or TA access was used in 50 (4.7%) and 90 (8.5%) patients respectively. Self-expanding Medtronic Evolut R valves were used in 84% of TS procedures, while balloon-expandable Edwards SAPIEN valves were used in all TA procedures. The TS group had a lower mean logistic EuroSCORE than the TA group (27.31 ± 19.44% vs 34.92 ± 19.61% *p* = 0.029). The TS approach was associated with a higher incidence of moderate postprocedural aortic regurgitation (12.5% vs 2.4%, *p* = 0.025). There was no significant difference in 30-day, 1-year or overall all-cause mortality.

**Conclusions:**

Both trans-subclavian and trans-apical access are viable approaches for patients requiring non-transfemoral TAVI. Differences in peri-procedural indices reflect the disparate patient populations and factors governing prosthesis choice, and short- and long-term mortality was similar.

## Introduction

Over the last two decades, the treatment of aortic valve stenosis has changed significantly with transcatheter aortic valve implantation (TAVI) becoming widely used in symptomatic elderly patients with high surgical risk. Conventional transfemoral (TF) access is generally considered the safest, with lower 1-year mortality compared with other approaches (16.4% vs. 24.8%) [1]. In addition, according to the PARTNER 2 trial, the advantage of TAVI over surgery was the greatest in the transfemoral approach [2]. Whilst this may, in part, reflect differences in co-morbidity, between 15 and 20% of patients are unsuitable for transfemoral TAVI [3] and an alternative delivery route is necessary. This is usually due to unfavourable iliofemoral or aortic anatomy. Several non-transfemoral access sites have been described, including trans-subclavian (TS), trans-apical (TA), direct aortic, trans-carotid, trans-caval and trans-venous (via the interatrial septum). When the TF approach is not possible, the choice of delivery is based upon the patient’s anatomy, availability of a particular valve system, operator preference and local expertise. Among non-transfemoral approaches, the TA approach seems to be the preferred method in most centres [4, 5]; however, the evidence supporting the use of one particular access over the other is limited. In addition, most of the data exploring this topic come from studies using first and second generation valves [1, 6].

To address this, we performed a national, retrospective observational study comparing the pre-operative characteristics, operative parameters, and postoperative outcomes and complications for all patients undergoing TS and TA TAVI for severe, symptomatic aortic stenosis in Scotland between January 2013 and March 2020.

## Methods

### Patient population

**Fig. 1:**
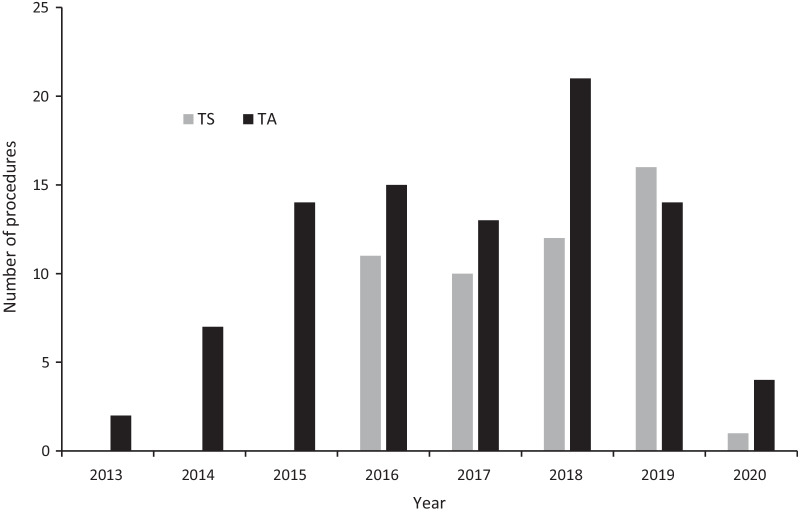
Number of TS and TA procedures performed each year

Transcatheter aortic valve implantation began in Scotland in 2012, and until 2018 the Royal Infirmary of Edinburgh was the sole TAVI centre in the country; there are now three. The Royal Infirmary of Edinburgh cardiothoracic surgical unit remains the only provider of non-transfemoral TAVI in Scotland. All cases are discussed at a multidisciplinary team (MDT) meeting, with determination of the access route made on the basis of the factors mentioned above. The initial prosthesis available for use was the SAPIEN balloon-expandable valve (Edwards Lifesciences, Irvine, CA; currently the SAPIEN 3) with subsequent introduction of the self-expanding Evolut R (Medtronic, Minneapolis, MN) in 2015 (Fig. 1). In cases where TF access is not possible, an alternative approach is considered with TS being preferred over TA (Table 1).

**Table 1:** Choice of access route

Factors favouring TS approach (predominantly Evolut R)	Factors favouring TA approach (exclusively SAPIEN)
Favourable subclavian artery anatomy	No apical left ventricular aneurysm or thrombus
Aortic annulus angulation < 70° (left subclavian) or < 30° (right subclavian)	No severe aortic annuluar calcification
No pedicled internal mammary coronary artery bypass graft	Low probability of native coronary artery occlusion (adequate coronary sinus width and coronary artery height)

TS = trans-subclavian; TA = trans-apical

Data for all patients during the study period was extracted from the National Institute of Cardiovascular Outcomes Research (NICOR) TAVI registry. Using this dataset, we identified all patients undergoing TS or TA TAVI at the Royal Infirmary of Edinburgh between January 2013 and March 2020. Baseline demographics, procedural characteristics and outcomes were obtained from medical record review. Missing values were not imputed. Mortality tracking was performed on 6th October 2020 using the Scottish Community Health Index (CHI) database. All procedural and outcome parameters were defined using the NICOR dataset version 4.09 definitions [7].

Statistical Package for Social Science (SPSS) version 25.0 [8] was used for statistical analysis. Continuous variables are presented as means ± SD or median with interquartile range (IQR), while categorical variables are presented as frequencies with percentages in brackets. Continuous variables were compared using independent samples 2-tailed student t-test and categorical variables using the chi-squared test or Fisher’s exact test as appropriate. Survival analysis was done using the Kaplan–Meier method with the log-rank test for comparison between groups. Univariate and multivariate Cox regression models were constructed with age, sex and access route chosen as covariates. A p-value of < 0.05 was considered statistically significant.

## Results

**Fig. 2:**
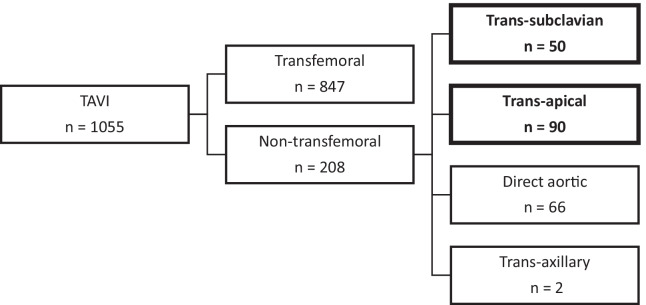
TAVI procedures performed in the Royal Infirmary of Edinburgh between January 2013 and March 2020, according to the access site used

Between January 2013 and March 2020, 1055 patients with symptomatic aortic valve disease underwent TAVI at the Royal Infirmary of Edinburgh. Overall, 208 patients (19.7%) were unsuitable for TF access. Of these, 50 (4.7%) and 90 (8.5%) underwent TAVI via a TS or TA approach (Fig. 2); These 140 patients formed the cohort for analysis.

### Population

This was an elderly, high-risk, predominantly Caucasian population; approximately half were female (Table 2). There was a high prevalence of vascular disease and other comorbidities, with consequently high EuroSCOREs, particularly in the TA group. This was the first TAVI procedure for all patients. The only significant difference between the TS and TA groups was the prevalence of previous cardiac surgery (46.7% vs. 18%, *p* < 0.001). In most cases, this was previous coronary artery bypass grafting (CABG).

**Table 2:** Baseline characteristics

Parameter	Overall (n = 140)	TS (n = 50)	TA (n = 90)	*p*-value
Demographics				
Age (mean ± SD)	79.07 ± 7.14	79.42 ± 7.84	78.88 ± 6.77	0.669
Men	73 (52.1%)	22 (44%)	51 (56.7%)	0.151
Ethnic origin (white)	139 (99.3%)	50 (100%)	89 (98.9%)	0.454
Weight (kg) (mean ± SD)	71.56 ± 19.1	73.06 ± 23.2	70.59 ± 16.1	0.478
Height (m) (mean ± SD)	164 ± 0.96	1.63 ± 0.10	1.64 ± 0.93	0.322
Risk factors				
Diabetes mellitus	31 (22.1%)	13 (26%)	18 (20%)	0.413
Current or ex-smoker	89 (63.6%)	31 (62%)	58 (64.4%)	0.855
Creatinine (μmol/l) (mean ± SD)	102.89 ± 44.020	103.22 ± 45.925	102.7 ± 43.187	0.947
On dialysis	1 (0.7%)	1 (2%)	0 (0%)	0.357
Previous MI	37 (26.4%)	10 (20%)	27 (30%)	0.234
History of pulmonary disease	46 (32.9%)	17 (34%)	29 (32.2%)	0.710
Severe liver disease	1 (0.7%)	0 (0%)	1 (1.1%)	1.000
History of neurological disease	27 (19.3%)	11 (12%)	16 (17.8%)	0.655
Extracardiac arteriopathy	116 (82.9%)	37 (74%)	79 (87.8%)	0.059
Poor mobility	29 (20.7%)	12 (24%)	17 (18.9%)	0.460
Extensive calcification of ascending aorta	30 (21.4%)	13 (26%)	17 (18.9%)	0.391
Logistic EuroSCORE (%)(mean ± SD)	32.21 ± 19.82	27.31 ± 19.44	34.92 ± 19.61	**0.029**
Previous interventions				
Previous cardiac surgery	51 (36.4%)	9 (18%)	42 (46.7%)	**0.001**
Balloon valvuloplasty prior to TAVI	8 (5.7%)	3 (6%)	5 (5.6%)	1.000
Previous TAVI	0 (0%)	0 (0%)	0 (0%)	NS
Previous PCI	30 (21.4%)	10 (20%)	20 (22.2%)	0.832
Clinical status				
Critical pre-operative status	73 (52.1%)	22 (44.0%)	51 (52.1%)	0.162
CCS Angina Status				
0	101 (72%)	36 (72%)	65 (72%)	1.000
I	22 (15.7%)	7 (14%)	15 (16.7%)	0.810
II	10 (7.1%)	6 (12%)	4 (4.4%)	0.167
III	7 (5%)	1 (2%)	6 (6.7%)	0.421
IV	0 (0%)	0 (0%)	0 (0%)	NS
NYHA dyspnoea status				
I	5 (3.6%)	2 (4%)	3 (3.3%)	1.000
II	4 (2.9%)	1 (2%)	3 (3.3%)	1.000
III	85 (60.7%)	26 (52%)	59 (65.6%)	0.149
IV	46 (32.9%)	21 (42%)	25 (27.8%)	0.094
Results of cardiac investigations				
Co-existing aortic regurgitation	8 (5.7%)	6 (12%)	2 (2.2%)	**0.025**
Co-existing mitral regurgitation	78 (55.7%)	29 (58%)	49 (54.4%)	0.725
One or more coronary vessels with > 50% diameter stenosis	76 (54.3%)	22 (44%)	54 (60%)	0.069
Aortic valve mean gradient (mmHg) (mean ± SD)	44.23 ± 14.91	43.91 ± 16.99	44.41 ± 13.72	0.857
Aortic valve peak gradient (mmHg) (mean ± SD)	75.38 ± 22.37	74.20 ± 25.37	76.07 ± 20.53	0.641
LV function				
Good (LVEF ≥ 50%)	81 (57.9%)	32 ( 64%)	49 (54.4%)	0.290
Fair (LVEF = 30–49%)	36 (25.7%)	10 (20%)	26 (28.9%)	0.314
Poor (LVEF < 30%)	23 (16.4%)	8 (16%)	15 (16.7%)	1.000

TS = trans-subclavian; TA = trans-apical; MI = myocardial infarction; PCI = percutaneous coronary intervention; LV = left ventricle; LVEF = left ventricular ejection fraction. Bold numbers indicate a significant difference between groups

### Procedural parameters

Intraoperative results are summarised in Table 3. The TA group consisted exclusively of SAPIEN valves, whereas the majority of TS cases used an Evolut R. All cases except for one TS case were performed under general anaesthetic. Transoesophageal echo was used routinely in all general anaesthetic cases. Balloon valvuloplasty before valve deployment was performed more often in the TS group (38.0% vs. 7.8%, *p* < 0.001), in keeping with differences in technique (antegrade versus retrograde prosthesis delivery) and prosthesis type. The procedure duration was similar in both groups.

**Table 3:** Intra-procedural parameters

Parameter	Overall (n = 140)	TS (n = 50)	TA (n = 90)	*p*-value
Urgent procedure	23 (16.4%)	12 (24%)	11 (12.2%)	0.095
Aortic balloon valvuloplasty before valve deployment	26 (18.6%)	19 (38.0%)	7 (7.8%)	** < 0.001**
Procedure time (min)(mean ± SD)	57.4 ± 27.59	63.6 ± 39.86	54.19 ± 17.62	0.073
Valve type				
Edwards SAPIEN 3	92 (65.7%)	6 (12%)	86 (95.6%)	
Medtronic Evolut R	42 (30%)	42 (84%)	0 (0%)	
Edwards SAPIEN 3 Ultra	2 (1.4%)	2 (4%)	0 (0%)	
Edwards SAPIEN XT	4 (2.9%)	0 (0%)	4 (4.4%)	

TS = trans-subclavian; TA = transapical. Bold numbers indicate a significant difference between groups

### Procedural outcomes

The procedural outcomes and in-hospital complications are summarised in Table 4. There was one instance of device failure, where the Evolut R capsule snapped and the device had to be removed. Immediate complications were rare. Post-dilatation was performed more frequently in TS cases (22.4% vs. 8.3% *p* = 0.034). Patients in the TS group were more likely to have moderate aortic regurgitation at the end of the procedure (12.5% vs. 2.4%, *p* = 0.006). No patients had severe aortic regurgitation at case completion. Vascular access site and access related complications were more common in the TS approach (12% vs. 4.6%, *p* = 0.105) but the difference was not statistically significant. The median length of hospital stay was similar between the two groups.

**Table 4:** Procedural outcomes, complications and mortality

Parameter	Overall (n = 140)	TS (n = 50)	TA (n = 90)	*p*-value
Aortic regurgitation at the end of procedure				
None (%)	69 (51.9%)	17 (35.4%)	52 (61.2%)	**0.006**
Mild (%)	56 (42.1%)	25 (52.1%)	31 (36.5%)	0.053
Moderate (%)	8 (6%)	6 (12.5%)	2 (2.4%)	**0.025**
Severe (%)	0 (0%)	0 (0%)	0 (0%)	NS
Valve malpositioning (migration) (%)	1 (0.7%)	1 (2.0%)	0 (0%)	0.363
Bail-out valve-in-valve (%)	1 (0.7%)	1 (2.0%)	0 (0%)	0.380
Post implantation balloon dilatation of implanted valve (%)	18 (13.5%)	11 (22.4%)	7 (8.3%)	**0.034**
Peri- and post procedural complications	12 (8.6%)	4 (8%)	8 (8.9%)	1.000
Permanent pacing post procedure (%)	8 (5.8%)	3 (6%)	5 (5.7%)	1.000
Vascular access site and access related complications (%)	10 (7.1%)	6 (12%)	4 (4.6%)	0.105
Acute Kidney Injury within 7 days of procedure (%)	16 (12.1%)	3 (6.3%)	13 (15.5%)	0.167
Length of hospital stay (days) (mean ± SD)	10.2 ± 10.7	10.7 ± 11.6	9.92 ± 10.3	0.682
Median (IQR)	6 (6)	6 (6.25)	7 (6)	
In-hospital mortality (%)		4 (8%)	5 (5.6%)	0.721
30-day mortality (%)		3 (6%)	5 (5.6%)	0.905
1-year mortality (%)		9 (18%)	14 (15.6%)	0.704
3-year mortality (%)		16 (32%)	24 (26.7%)	0.302
Follow-up time (days) (mean ± SD)		680 ± 470	1006 ± 686	**0.003**

TS = trans-subclavian; TA = trans-apical; IQR = interquartile range. Bold numbers indicate a significant difference between groups

### Survival

**Fig. 3:**
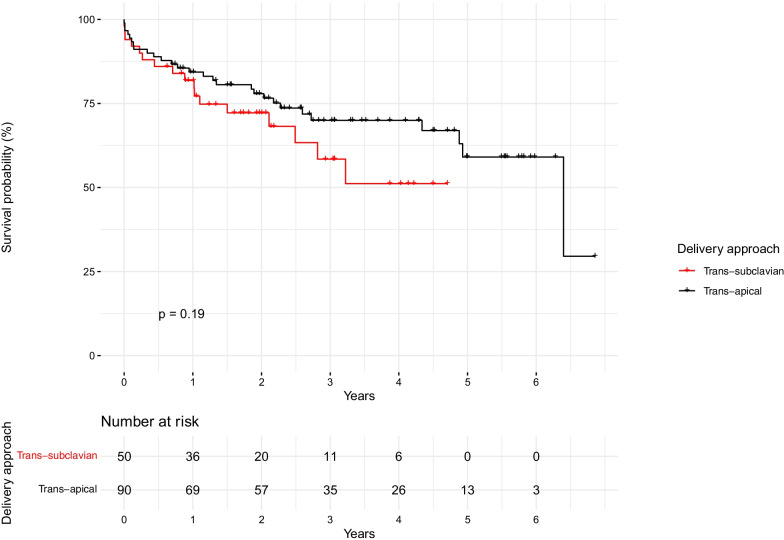
Overall all-cause mortality according to delivery approach

Follow-up time was significantly longer in the TA group. Mortality was similar between groups (Table 4, Fig. 3). On multivariable Cox regression analysis, access site was not associated with mortality after adjusting for age and sex (Table 5).

**Table 5:** Predictors of all-cause mortality

	Univariate			Multivariate		
Variables	HR	95% CI	*p*-value	HR	95% CI	*p*-value
TS approach	0.663	0.355–1.235	0.195	0.678	0.360–1.280	0.231
Age (per year)				1.002	0.963–1.043	0.904
Sex (males)				0.880	0.487–1.590	0.673

TS = trans-subclavian; CI = confidence interval; HR = hazard ratio

## Discussion

In this national observational study, we compared outcomes of TS and TA TAVI procedures. Although there was a higher prevalence of moderate post-procedural aortic regurgitation in the TS group, there was no difference in vascular and access site complications, length of stay or early or late mortality. These data confirm that both access sites are reasonable in selected patients at centres with the requisite experience, with consideration of individual patient factors and anatomy being paramount in each institution’s heart team decision algorithm.

### Clinical outcomes and complications

In our study, we did not find any differences in the device success rates between the two methods, which confirms the findings from a multicentre trial by Ciuca et al. [9]. One of the key differences between previous studies and our study was the valve delivery systems used. Trials included in two published meta-analyses [1, 6] almost exclusively used Edwards SAPIEN and Medtronic CoreValve systems. Procedures included in our study used newer, third-generation valves, mostly Edwards SAPIEN 3 and Medtronic Evolut R, which could be contributing to the differences in results. However, this must be interpreteted with caution given that Evolut R was used predominantly for TS and Edwards SAPIEN 3 exclusively for TA approach. This is the most salient point when contextualising our data. The choice of access site and prosthesis is governed by patient factors, such as comorbidities and anatomy, as well as institutional factors, such as device availability and operator experience. Most of the between-group differences here are explained by anatomy (eg. more CABG in TA group due to use of pedicled internal mammary grafts) and prosthesis type (eg. more immediate post-TAVI aortic regurgitation with self-expanding valves).

Moderate aortic regurgitation at the end of the procedure was more prevalent in the TS group than in TA (12.5% vs. 2.4% *p* = 0.025). This is different from ﻿previously reported findings by Taramasso et al. which demonstrated that no significant differences were observed between TS and TA with regards to postprocedural aortic regurgitation [10]. This could be explained by the fact that the majority of TS patients received a self-expandable Medtronic Evolut R valve. Self-expandable devices seem to be associated with higher rates of postprocedural aortic regurgitation than balloon-expandable systems [11]. Importantly, we did not have data on subsequent outpatient post-TAVI aortic regurgitation, at which time there may be reduced paravalvular leak as annular sealing improves with expansion of the nitinol Evolut R valve frame.

Another important finding is the lack of difference in permanent pacemaker implantation rates between two access sites. It was previously shown that early pacemaker implantation was more frequent after TS procedures than TA [6]. It was also reported that pacemaker implantation rates tend to be higher with Evolut R valves compared with SAPIEN 3 [12].

Vascular access site and access-related complications were not significantly different between two groups. This is in keeping with a recent multicentre study that reported the rate of vascular complications among TS and TA as 10% and 9.9%, respectively [9]. Additionally, another meta-analysis showed indirectly that the TS method was associated with a decrease in vascular complications compared with TA [13].

### Mortality

The meta-analysis by Chandrasekhar et al. [1] and data from the UK TAVI registry [14, 15], both suggest that the TS approach is associated with a lower mortality rate compared with TA. A more recent meta-analysis by Takagi et al. [6] also claims that early all-cause mortality is lower in TS than TA groups; however, at mid-term, the mortality was equivalent between TS and TA. Data from the FRANCE-2 TAVI registry [4] seem to be the only source suggesting that the TS approach is associated with increased late mortality. In addition, two Italian studies reported no significant differences in mortality between TS and TA access sites [9, 16]. Our results are congruent with these data. It is also important to note that the EuroSCORE was significantly higher in the TA group. However, the EuroSCORE was not developed for risk stratification in TAVI. Interestingly, despite the seemingly more invasive nature of the TA procedure, our data suggest no difference in mortality or length of hospital stay.

As described previously in the methods section, in cases when TF approach was not possible, the TS approach was favoured by the MDTs in our centre, and TA was only chosen in cases when TS was not possible. In spite of this, our results demonstrate that the mortality of the TA approach was equivalent.

### Limitations

Our study has several limitations. Most obviously and importantly, this study was a single centre, retrospective observational study with a relatively small sample size. Consequently, there are multiple unmeasured confounders. Most pertinently, as access sites were chosen on a clinical basis, there is inherent selection bias. These data cannot, therefore, be used to infer any causal relationship between access route and clinical outcomes. Rather, they must be interpreted in the context of a single centre—albeit relatively high volume—registry. Our sample size further limits interpretation of the data, since prosthesis models, temporal trends in patient selection (from inoperable to high or intermediate risk) and local accrued experience may play a role in procedural and clinical outcomes. Amongst the measured variables, the most obvious difference is the choice of prosthesis, with the Evolut R being unsuitable for TA delivery. We were not able to collect data on quality of life or symptomatic improvement, which is important as the indication for TAVI is symptom improvement rather than prognosis; indeed the former may be a more relevant consideration than the latter in some patients. Finally, we did not have access to clinical outcome data other than all-cause mortality.

## Conclusions

In this national retrospective study of patients undergoing TS or TA access for TAVI, length of stay and mortality did not differ significantly between patients underoing TAVI via TS or TA access. Either approach is feasible in the context of an appropriate heart team decision algorithm, accounting for patient and institutional factors.

## Abbreviations

CABG: Coronary artery bypass graft
CHI: Community Health Index
CI: Confidence interval
HR: Hazard ratio
IQR: Interquartile range
LV: Left ventricle
LVEF: Left ventricular ejection fraction
MDT: Multidisciplinary team
MI: Myocardial infarction
NICOR: National Institute for Cardiovascular Outcomes Research
PCI: Percutaneous coronary intervention
SD: Standard deviation
SPSS: Statistical Package for Social Science
TA: Trans-apical
TAVI: Transcatheter aortic valve implantation
TF: Trans-femoral
TS: Trans-subclavian

## References

[1] Chandrasekhar J, Hibbert B, Ruel M, Lam B-K, Labinaz M, Glover C (2015). Transfemoral vs non-transfemoral access for transcatheter aortic valve implantation: a systematic review and meta-analysis. Can J Cardiol.

[2] Leon MB, Smith CR, Mack MJ, Makkar RR, Svensson LG, Kodali SK (2016). Transcatheter or surgical aortic-valve replacement in intermediate-risk patients. N Engl J Med.

[3] Grover FL, Vemulapalli S, Carroll JD, Edwards FH, Mack MJ, Thourani VH (2017). 2016 annual report of the society of thoracic surgeons/American College of Cardiology Transcatheter valve therapy registry. J Am Coll Cardiol.

[4] Gilard M, Eltchaninoff H, Donzeau-Gouge P, Chevreul K, Fajadet J, Leprince P (2016). Late outcomes of transcatheter aortic valve replacement in high-risk patients: the FRANCE-2 registry. J Am Coll Cardiol.

[5] Moat NE, Ludman P, de Belder MA, Bridgewater B, Cunningham AD, Young CP (2011). Long-Term Outcomes After Transcatheter Aortic Valve Implantation in High-Risk Patients With Severe Aortic Stenosis: The U.K. TAVI (United Kingdom Transcatheter Aortic Valve Implantation) Registry. J Am Coll Cardiol.

[6] Takagi H, Hari Y, Nakashima K, Kuno T, Ando T (2019). Comparison of early and midterm outcomes after transsubclavian/axillary versus transfemoral, transapical, or transaortic transcatheter aortic valve implantation. Hear Lung.

[7] National Institute of Cardiovascular Outcomes Research. Transcatheter Aortic Valve Implantation (TAVI) Dataset [Internet]. 2013 [cited 2021 Mar 16]. Available from: https://www.nicor.org.uk/national-cardiac-audit-programme/datasets/

[8] IBM Corp. IBM SPSS Statistics for Macintosh version 25.0. 2017.

[9] Ciuca C, Tarantini G, Latib A, Gasparetto V, Savini C, Di Eusanio M (2016). Trans-subclavian versus transapical access for transcatheter aortic valve implantation: a multicenter study. Catheter Cardiovasc Interv.

[10] Taramasso M, Maisano F, Cioni M, Denti P, Godino C, Montorfano M (2011). Trans-apical and trans-axillary percutaneous aortic valve implantation as alternatives to the femoral route: short- and middle-term results✩. Eur J Cardio-Thoracic Surg.

[11] Eric VB, Françis J, Sophie S, André V, Bernard I, Jean D (2014). Postprocedural aortic regurgitation in balloon-expandable and self-expandable transcatheter aortic valve replacement procedures. Circulation.

[12] Pierre D, Arnaud B, Julien H, Thibaud L, Christophe SE, Leslie G-G (2020). Impact of Sapien 3 balloon-expandable versus evolut R self-expandable transcatheter aortic valve implantation in patients with aortic stenosis. Circulation.

[13] Garcia DC, Benjo A, Cardoso RN, Macedo FYB, Chavez P, Aziz EF (2014). Device stratified comparison among transfemoral, transapical and transubclavian access for transcatheter aortic valve replacement (TAVR): a meta-analysis. Int J Cardiol.

[14] Fröhlich GM, Baxter PD, Malkin CJ, Scott DJA, Moat NE, Hildick-Smith D (2015). Comparative survival after transapical, direct aortic, and subclavian transcatheter aortic valve implantation (Data from the UK TAVI Registry). Am J Cardiol.

[15] Ludman PF (2019). UK TAVI registry. Heart.

[16] Adamo M, Fiorina C, Curello S, Maffeo D, Chizzola G, Di Matteo G (2015). Role of different vascular approaches on transcatheter aortic valve implantation outcome: a single-center study. J Cardiovasc Med.

